# Supplementary material for: Hetero-Bimetallic Ferrocene-Containing Zinc(II)-Terpyridyl-Based Metallomesogen: Structural and Electrochemical Characterization
Source: Materials (Basel). 2023 Feb 27;16(5):1946. doi: 10.3390/ma16051946 (PMC10004155; doi:10.3390/ma16051946)
Supplement: Supplementary file 1 [file materials-16-01946-s001.zip › materials-2234067-supplementary.pdf]

Supplementary Materials

for

Hetero-Bimetallic Ferrocene-Containing Zinc(II)-Terpyridyl-Based Metallomesogen: Structural and Electrochemical Characterization

Evelyn Popa [1], Adelina A. Andelescu [1], Sorina Ilies (b. Motoc) [1], Alexandru Visan [1], Carmen Cretu [1], Francesca Scarpelli [2], Alessandra Crispini [2], Florica Manea [3,*] and Elisabeta I. Szerb [1,*]

[1] “Coriolan Drăgulescu” Institute of Chemistry, Romanian Academy, 24 Mihai Viteazu Bvd., 300223 Timisoara, Romania
[2] MAT-INLAB (Laboratorio di Materiali Molecolari Inorganici), Dipartimento di Chimica e Tecnologie Chimiche, Universitá della Calabria, Arcavacata, 87036 Rende, Italy
[3] Department of Applied Chemistry and Engineering of Inorganic Compounds and Environment, Politehnica University of Timisoara, Bvd. Vasile Parvan No. 6, 300223 Timisoara, Romania
* Correspondence: florica.manea@upt.ro (F.M.); eszerb@acad-icht.tm.edu.ro (E.I.S.)

Experimental section. Synthesis.

FT-IR spectra of Zn/Fe and Zn metallomesogens**. Figure S1**.

^{1}H NMR spectra of complex Zn/Fe. **Figure S2**.

TGA and DTG traces of complex Zn/Fe. **Figure S3**.

Electrochemical studies. **Figure S4**. Effect of the scan rate on CVs recorded at Zn_CNT, Fc_CNT and Zn/Fe_CNT paste electrodes

1. Experimental section. Synthesis.

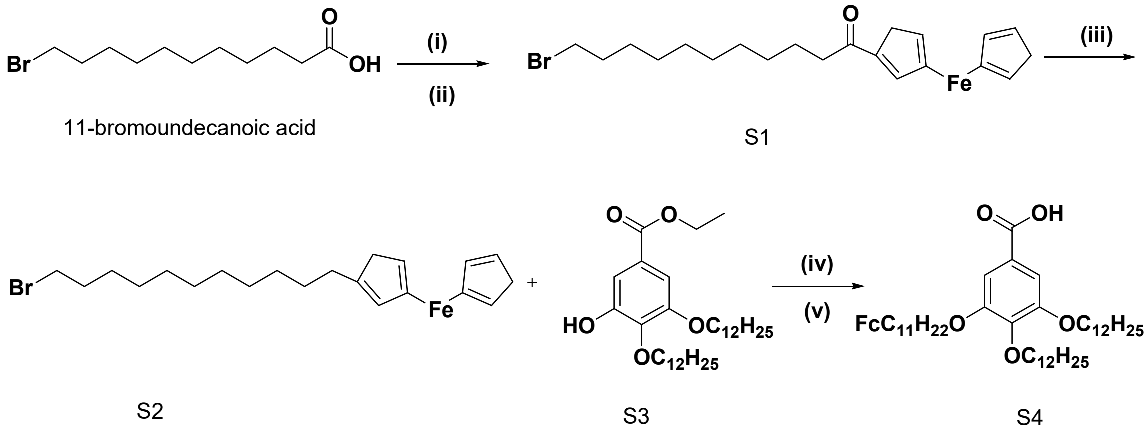

Scheme S1. (i) $SOCl_2$, toluene, 70°C, overnight; (ii) ferrocene, $AlCl_3$, DCM, 0°C, then r.t overnight; (iii) $AlCl_3$, $NaBH_4$, THF, 0°C, then r.t overnight; (iv) K_2CO_3, DMF, 80°C, 24 h; (v), KOH, EtOH, ΔT.

Synthesis of S1

In a three necked round bottomed flask was added under argon thionyl chloride (5.90 g, 49.57 mmol) over a solution of 11-bromoundecanoic acid (3.00 g, 11.31 mmol) in toluene (15 mL). The reaction mixture was stirred at 70°C overnight, cooled to r.t and then taken to dryness under reduced pressure. The excess of thionyl chloride was eliminated by repeated addition of DCM followed by evaporation of the solvent. The residue was then dissolved in 50 mL dry DCM and was added dropwise (during 1 hour) over a cooled mixture of ferrocene (2.53 g, 13.58 mmol) and $AlCl_3$ (1.81 g, 13.58 mmol) in 15 mL dry DCM. After the addition of the acyl chloride the suspension was allowed to reach room temperature and stirred overnight, after which the reaction mixture was quenched with ice cold water (cca 150 mL). The organic layer was separated and the aqueous phase was extracted with DCM (3 x 100 mL), then the combined organic layers were washed with water and brine, dried over anhydrous Na_2SO_4, filtered and taken to dryness. The crude product was purified by column chromatography (SiO_2/hexane:AcOEt = 9:1), yielding the desired compound as a dark orange solid (4.26 g, 9.83 mmol, 87%). $C_{21}H_{29}BrFeO$ (433.21 g·mol^{-1}). ^{1}H NMR (300 MHz, Chloroform-*d*, δ/ppm): 4.78 (t, ^{4}J = 1.9 Hz, 2H), 4.49 (t, ^{4}J = 2.0 Hz, 2H), 4.19 (s, 5H), 3.40 (t, ^{3}J = 6.9 Hz, 2H), 2.69 (t, ^{3}J =7.4 Hz, 2H), 1.84 (q, ^{3}J =7.1 Hz, 2H), 1.70 (q, ^{3}J =7.3 Hz, 2H), 1.45 – 1.23 (overlapped peaks, 12 H). FT-IR (KBr, cm^{-1}): $\nu_{C\text{-}H,\,Fc}$ (3128 – 3087), $\nu_{CH_2,as}$ (2917), $\nu_{CH_2,s}$ (2850), ν_{CO} (1659), out of-plane vibration of Fc cyclopentadiene (1106, 1002, 825), $\nu_{ring\text{-}metal,\,Fc}$ (481).

Synthesis of S2

A solution of S1 (3.00 g, 7.12 mmol) in 50 mL dry THF was added dropwise (during 1 hour) under argon, over a cooled mixture of $AlCl_3$ (3.80 g, 28.49 mmol) and $NaBH_4$ (2.15 g, 56.96 mmol) in 35 mL THF. The reaction mixture was allowed to reach room temperature and was stirred overnight, after which the reaction mixture was quenched with ice cold water (cca 150 mL). The organic layer was separated and the aqueous phase was extracted with chloroform (3 x 100 mL), then the combined organic layers were washed with water and brine, dried over anhydrous Na_2SO_4, filtered and taken to dryness. The crude product was purified by column chromatography (SiO_2/ hexane:AcOEt = 20:1), yielding the desired compound as an orange solid (2.57 g, 86 %). $C_{21}H_{31}BrFe$ (419.22 g·mol^{-1}). ^{1}H NMR (300 MHz, Chloroform-*d*, δ/ppm): 4.08 (overlapped peaks, 9H), 3.44 (t, ^3J = 6.9 Hz, 2H), 2.33 (m, 2H), 1.83 (m, 2H), 1.30 – 1.56 (overlapped peaks, 14 H). FT-IR (KBr, cm^{-1}): $\nu_{C-H,Fc}$ (3095), $\nu_{CH_2,as}$ (2923), $\nu_{CH_2,s}$ (2849), out of-plane vibration of Fc cyclopentadiene (1103, 998, 818), $\nu_{ring-metal,\ Fc}$ (496).

Synthesis of S3

Ethyl 3,4,5-trihydroxybenzoate (10.0 g, 50.46 mmol) and K_2CO_3 (41.85 g, 302.77 mmol) in 200 mL cyclohexanone were mechanically stirred at 65ºC for 45 minutes, then 1-bromododecane (41.50 g, 40 mL, 166.52 mmol) was quickly added. The reaction mixture was stirred at 90ºC for 18 hours, then cooled to room temperature, filtered over cellite, and taken to dryness under reduced pressure. The residue was dissolved in DCM, washed with water (3 x 150 mL) and brine (1 x 100 mL), then the organic phase was dried over anhydrous Na_2SO_4, filtered and taken to dryness. The pure product was obtained by column chromatography (SiO_2/chloroform:hexane = 1:1) as a fluffy white solid (5.35 g, 25%). $C_{33}H_{58}O_5$ (534.81 g·mol^{-1}). ^{1}H NMR (300 MHz, Chloroform-*d*, δ/ppm): 7.30 (d, ^4J = 1.8 Hz, 1H), 7.18 (d, ^4J = 1.9 Hz, 1H), 5.87 (s broad, 1H, -O*H*), 4.17 (t, ^3J = 6.7 Hz, 2H, -OC*H*2-), 4.04 (t, ^3J = 6.4 Hz, 2H, -OC*H*2-), 4.09 (m, 2H, -COOC*H*2-), 1.79 (overlapped peaks, 4H), 1.56 – 1.18 (overlapped peaks, 38 H), 0.89 (t, ^3J = 6.6 Hz, 6H, -CH_2CH_3). FT-IR (KBr, cm^{-1}): ν_{O-H} (3466), $\nu_{CH_2,as}$ (2920), $\nu_{CH_2,s}$ (2851), $\nu_{C=O}$ (1700), ν_{C-O} (1231, 1106),

Synthesis of S4

First step

A mixture of ethyl 3,4-bis(dodecyloxy)-5-hydroxybenzoate, S3 (2.00 g, 3.74 mmol) and K_2CO_3 (1.59 g, 11.50 mmol) in 10 mL DMF was heated to 65°C, then S2 (1.93 g, 4.58 mmol) in 15 mL DMF was quickly added to the mixture. The reaction mixture was stirred at 80°C for 24 hours, cooled to room temperature and filtered over celite. The mixture was taken to dryness, then the residue was dissolved in 100 mL DCM and washed with water (3x) and brine, dried over anhydrous Na_2SO_4, filtered and dried under reduced pressure. The crude product was purified by column chromatography (SiO_2/hexane:AcOEt = 9:1), yielding the desired compound as an orange solid (2.03 g, 62.2%). $C_{54}H_{88}FeO_5$ (873.12 g·mol^{-1}). ^{1}H NMR (300 MHz, Chloroform-*d*, δ/ppm): 7.30 (s, 2H), 4.37 (q, ^{3}J=7.1 Hz, 2H, -COOCH_2-), 4.12 - 4.01 (overlapped peaks, 15H), 2.33 (m, 2H, -OCH_2-), 1.85 – 1.79 (overlapped peaks, 6 H), 1.59 – 1.29 (overlapped peaks, 55 H), 0.88 (t, J = 6.5 Hz, 6H, -CH$_2$CH_3). FT-IR (KBr, cm^{-1}): $\nu_{C\text{-}H,Fc}$ (3097), $\nu_{CH2,as}$ (2925), $\nu_{CH2,s}$ (2854), $\nu_{C=O}$ (1722), $\nu_{C\text{-}O}$ (1218, 1117), out of-plane vibration of Fc cyclopentadiene (1020, 999, 819), $\nu_{ring\text{-}Fe, Fc}$ (485).

Second step.

The intermediate product obtained in the first step (2.00 g, 2.30 mmol) was dissolved in the minimum amount of THF and the 50 mL EtOH and KOH (0.26 g, 4.6 mmol) were added; the reaction mixture was stirred under reflux until the total hydrolysis of the intermediate (monitored by TLC). The reaction mixture was cooled to room temperature and concentrated under reduced pressure; 50 mL of water was added and the mixture was acidified with HCl 6M to pH = 6, when a yellow precipitate formed. The product was filtered, washed with water and dried, giving the final product as yellow waxy solid (1.5 g, 77%). $C_{52}H_{84}FeO_5$ (845.07 g·mol^{-1}). ^{1}H NMR (300 MHz, Chloroform-*d*, δ/ppm): 7.34 (s, 2H), 4.11 – 4.02 (overlapped peaks, 15H), 2.33 (m, 2H, -OCH_2-), 1.88 – 1.72 (overlapped peaks, 6 H), 1.54 – 1.29 (overlapped peaks, 52 H), 0.90 (t, J = 6.5 Hz, 6H, -CH$_2$CH_3). FT-IR (KBr, cm^{-1}): $\nu_{C\text{-}H,Fc}$ (3097), $\nu_{CH2,as}$ (2921), $\nu_{CH2,s}$ (2851), $\nu_{C=O}$ (1680), $\nu_{C\text{-}O}$ (1234, 1115), out of-plane vibration of cyclopentadiene (999, 819), $\nu_{ring\text{-}Fe, Fc}$ (483).

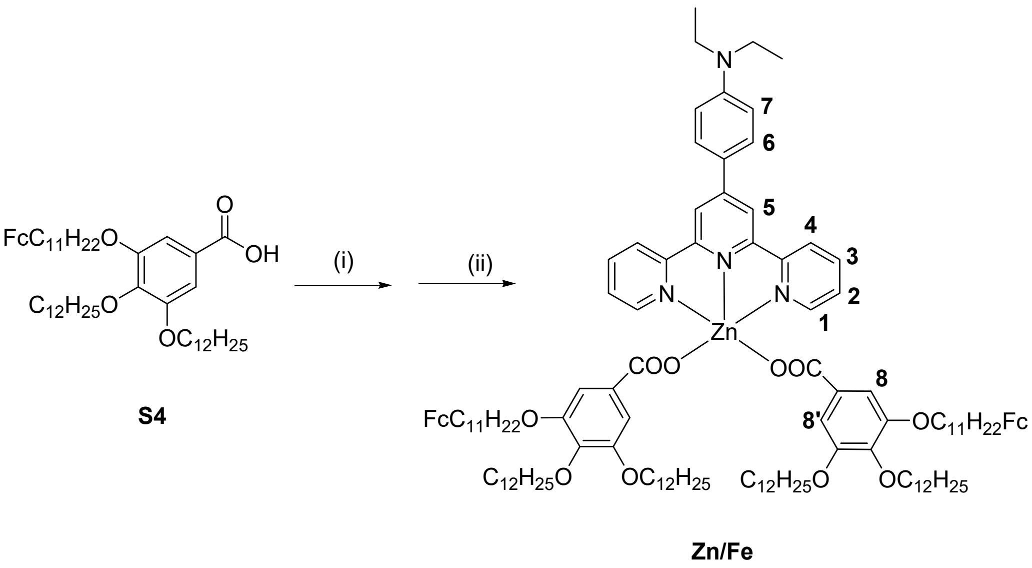

Scheme S2. Synthesis of complex Zn/Fe and NMR proton labelling. (i) NaOH, 30 min, THF, EtOH, then $ZnCl_2$ in EtOH, r.t., 24h; (ii) $tpyNEt_2$, $CHCl_3$, MeOH, 24h.

Synthesis of Zn/Fe

To a solution of S4 (0.200 g, 0.237 mmol) in 50 mL mixture of THF/EtOH = 1:50 (v/v) was added an aqueous solution of NaOH 10% (0.0095 g, 0.237 mmol). The reaction mixture was stirred at room temperature for 30 minutes, then $ZnCl_2$ (0.016 g, 0.119 mmol) dissolved in the minimum amount of EtOH was added dropwise. After 24 hours the solvent was removed under reduced pressure to give a pale orange residue, which was used without any purifications in the next step. The residue was dissolved in 5 mL of $CHCl_3$, a solution of 4-(4-N,N-diethylbenzenamine)-2,6-di(pyridine-2-yl)pyridine (0.045g, 0.119 mmol) in MeOH/$CHCl_3$ was added dropwise resulting in a brown solution which was stirred at r.t for 24 hours. After that the solvents were removed under reduced pressure. Zn/Fe was obtained as a yellow solid as following: firstly, the residue was recrystallized from Et_2O/hexane, the precipitate was filtered off, and the mother liquor taken to dryness. Then the residue was redissolved in hexane and precipitated out of ethanol. The complex was obtained as yellow solid (0.178 g, 70%). Anal. Calcd. for $C_{129}H_{190}Fe_2N_4O_{10}Zn \cdot 1.0\ H_2O$ (2152.00 g·mol^{-1}): C, 72.00; H, 8.99; N, 2.60. Found: C, 72.08; H, 9.12; N, 2.71%. AAS: Zn% calcd.: 3.15, found: 3.08. IR (KBr, cm^{-1}): 2924 ($\nu_{as,(\text{-CH2-})}$), 2849 ($\nu_{s,(\text{-CH2-})}$), 1615 ($\nu_{as\ COO}$-), 1356 ($\nu_{s\ COO}$-): Δ = 259 cm^{-1}, 1002, 924, 486 cm^{-1} (ν_{Fc}); ^{1}H NMR (500 MHz, Methylene Chloride-*d*2, δ/ppm) 8.77 (dd, ^3J = 4.9, ^4J = 1.7 Hz, 2H, H^1), 8.20 (s, 2H, H^5), 7.98 (d, ^3J = 8.1 Hz, 2H, H^4), 7.67 (d, ^3J = 8.6 Hz, 2H, H^6), 7.50 (t, ^3J = 7.4 Hz, 2H, H^3), 7.26 (m, 2H, H^2), 7.21 (s, 4H, H$^{8,8'}$), 6.33

(d, ^3J = 8.6 Hz, 2H, H^7), 3.98 (s, 10H, H^{Fc}), 3.95 (m, 4H, H^{Fc}), 3.92 (m, 4H^{Fc}), 3.85 (t, ^3J = 6.5 Hz, 8H, -OCH_2-), 3.82 (t, J = 6.6 Hz, 4H, -OCH_2-), 3.25 (m, 4H, -NCH_2-), 2.20 (m, 4H, -CH_2-Fc), 1.75 – 1.10 (overlapped peaks, 122 H), 0.79 (t, J = 6.9 Hz, 12H). ^{13}C NMR (126 MHz, CD_2Cl_2, δ/ppm) 171.57, 152.74, 152.34, 149.46, 148.73, 148.62, 148.48, 139.90, 138.78, 131.61, 128.39, 125.57, 121.21, 121.02, 116.80, 111.24, 107.75, 89.60, 73.21, 68.93, 68.38, 68.02, 66.91, 53.87, 53.66, 53.44, 53.22, 53.01, 44.29, 31.96, 31.95, 31.16, 30.36, 29.77, 29.75, 29.73, 29.71, 29.67, 29.63, 29.59, 29.50, 29.48, 29.41, 29.39, 26.16, 26.14, 22.71, 13.91, 12.51.

FT-IR (KBr, cm^{-1}): $\nu_{C\text{-}H,Fc}$ (3087), $\nu_{CH_2,as}$ (2923), $\nu_{CH_2,s}$ (2852), $\nu_{C=O}$ (1594), $\nu_{C\text{-}O}$ (1218, 1114), out of-plane vibration of Fc cyclopentadiene (1002, 924), $\nu_{ring\text{-}Fe,\,Fc}$ (486).

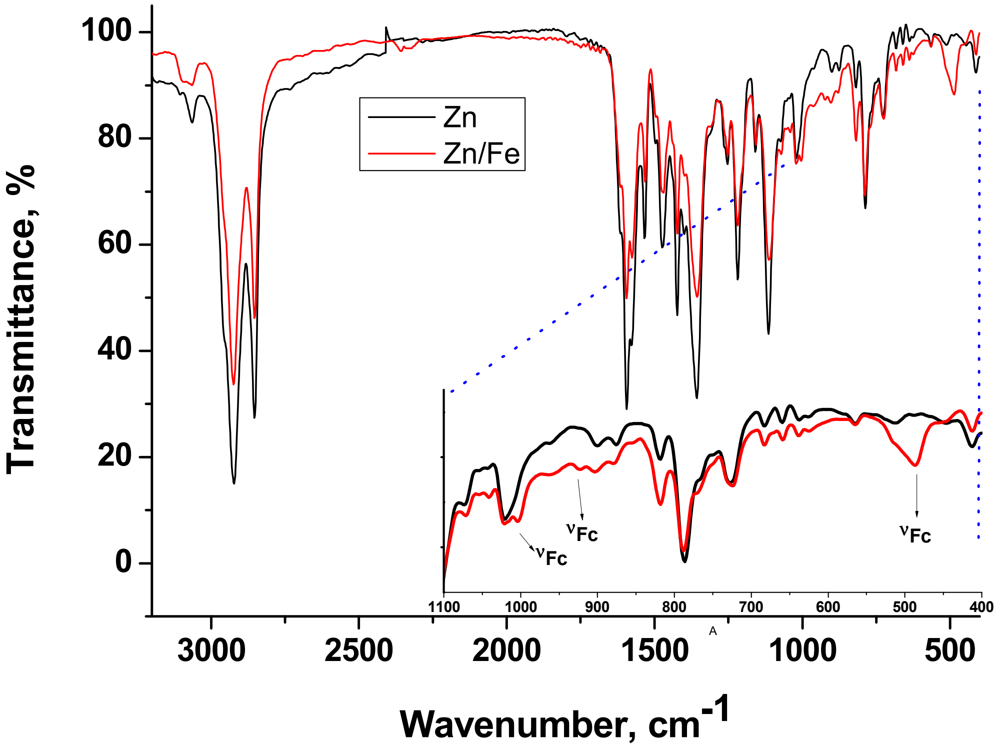

Figure S1. FT-IR spectra of complexes Zn/Fe and Zn metallomesogens. In the inset the region in which the vibrational bands of the ferrocene are observed is presented.

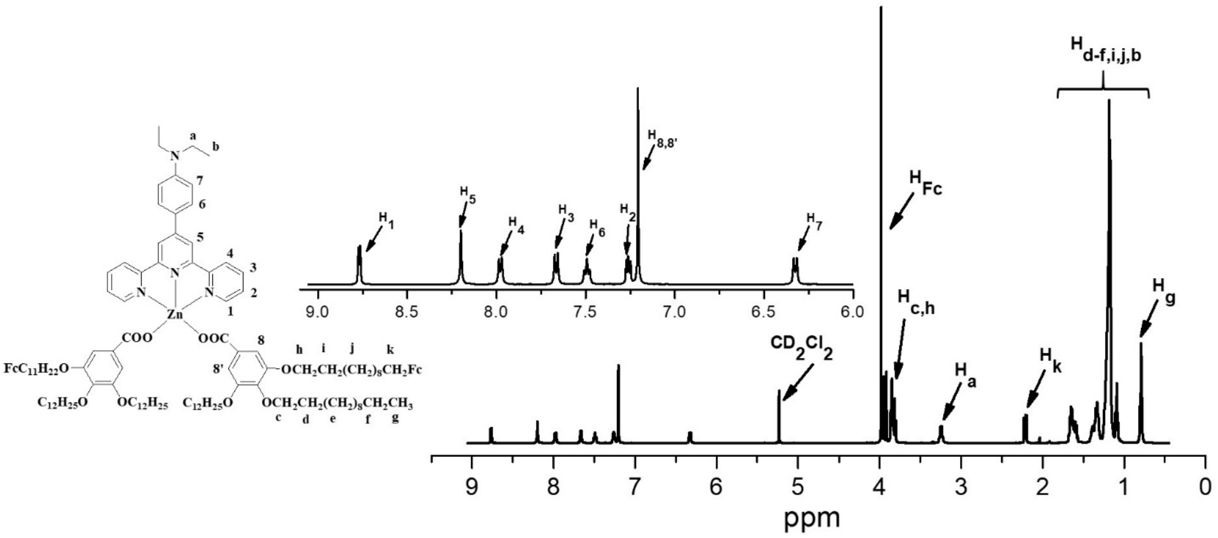

Figure S2. ^{1}H NMR spectra of complex Zn/Fe.

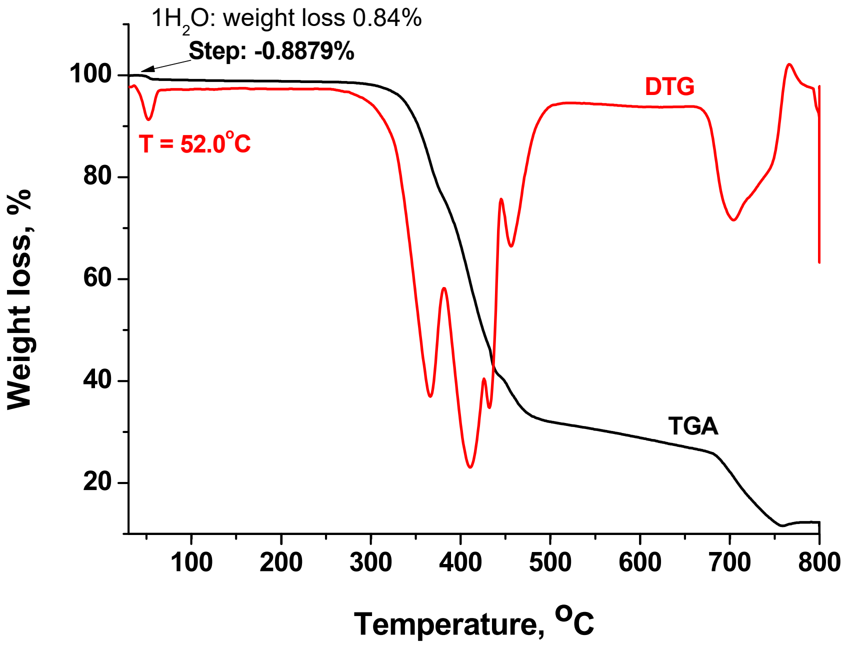

Figure S3. TGA and DTG traces of complex Zn/Fe: Loss of 1.0 molecule of water under 100°C. Weight loss from degradation becomes significant above ca. 300°C and reaches 5% at $T_{5\%}$ = 342°C.

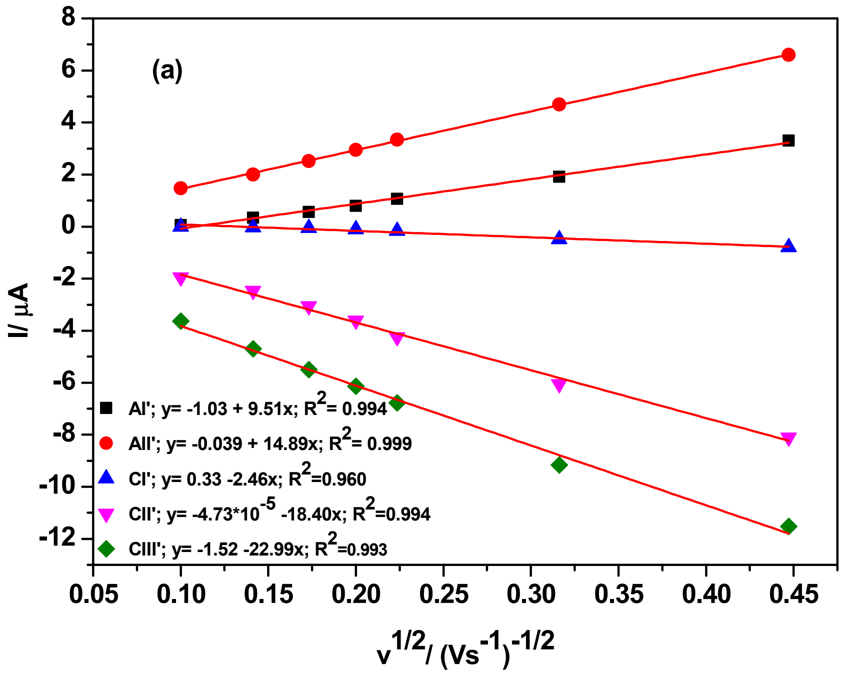

(a)
AI'; y= -1.03 + 9.51x; R2= 0.994
AII'; y= -0.039 + 14.89x; R2= 0.999
CI'; y= 0.33 -2.46x; R2=0.960
CII'; y= -4.73*10-5 -18.40x; R2=0.994
CIII'; y= -1.52 -22.99x; R2=0.993
I/ µA
v1/2/ (Vs-1)-1/2

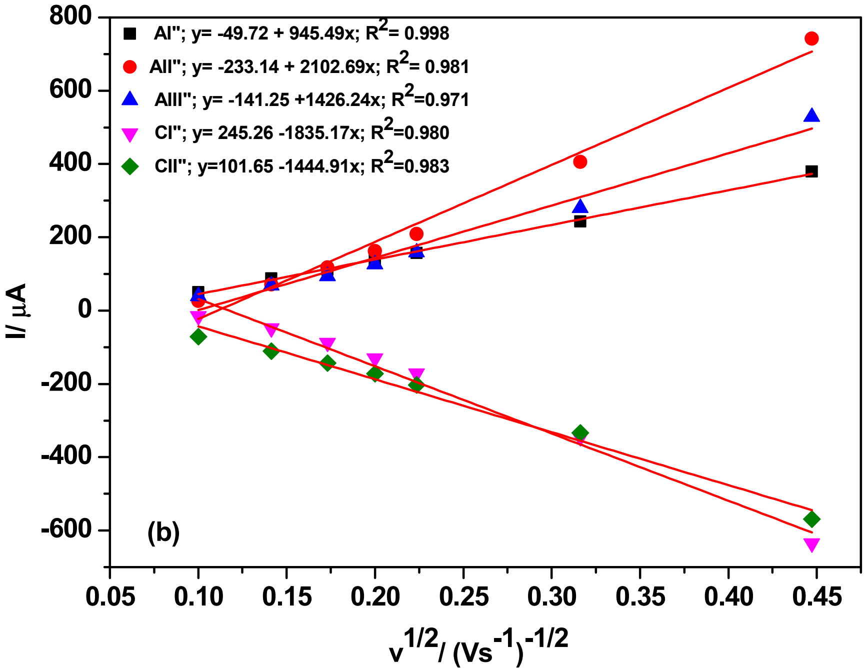

AI"; y= -49.72 + 945.49x; R2= 0.998
AII"; y= -233.14 + 2102.69x; R2= 0.981
AIII"; y= -141.25 +1426.24x; R2=0.971
CI"; y= 245.26 -1835.17x; R2=0.980
CII"; y=101.65 -1444.91x; R2=0.983
(b)
I/ µA
v1/2/ (Vs-1)-1/2

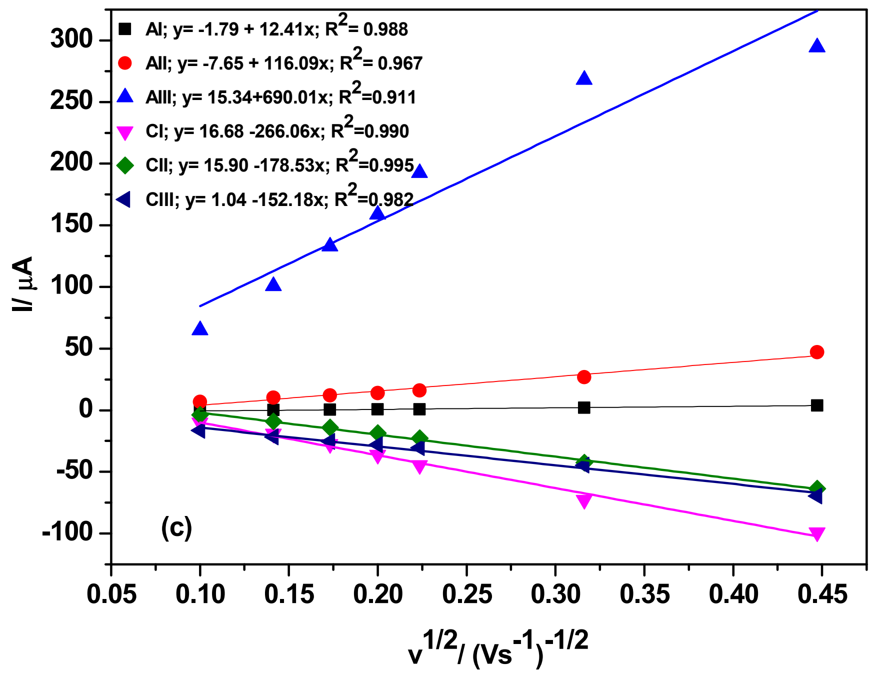

Figure S4. Linear dependence of the peaks currents vs. square root of the scan rate for: (a) Zn_CNT paste electrode; (b) Fc_CNT paste electrode; (c) Zn/Fe_CNT paste electrode
